# Fast Census of Moth Diversity in the Neotropics: A Comparison of Field-Assigned Morphospecies and DNA Barcoding in Tiger Moths

**DOI:** 10.1371/journal.pone.0148423

**Published:** 2016-02-09

**Authors:** Mauricio M. Zenker, Rodolphe Rougerie, José A. Teston, Michel Laguerre, Marcio R. Pie, André V. L. Freitas

**Affiliations:** 1 Departamento de Biologia Animal, Instituto de Biologia, Universidade Estadual de Campinas, Campinas-SP, Brazil; 2 Muséum national d’Histoire Naturelle, Sorbonne Universités, Institut de Systématique, Evolution, Biodiversité (ISYEB), UMR7205 – CNRS, UPMC, EPHE, Paris, France; 3 Laboratório de Estudos de Lepidópteros Neotropicais and Programa de Pós-Graduação em Recursos Naturais da Amazônia, Universidade Federal do Oeste do Pará, Santarém, PA, Brazil; 4 Departamento de Zoologia, Universidade Federal do Paraná, Curitiba-PR, Brazil; Tuscia University, ITALY

## Abstract

The morphological species delimitations (*i*.*e*. morphospecies) have long been the best way to avoid the taxonomic impediment and compare insect taxa biodiversity in highly diverse tropical and subtropical regions. The development of DNA barcoding, however, has shown great potential to replace (or at least complement) the morphospecies approach, with the advantage of relying on automated methods implemented in computer programs or even online rather than in often subjective morphological features. We sampled moths extensively for two years using light traps in a patch of the highly endangered Atlantic Forest of Brazil to produce a nearly complete census of arctiines (Noctuoidea: Erebidae), whose species richness was compared using different morphological and molecular approaches (DNA barcoding). A total of 1,075 barcode sequences of 286 morphospecies were analyzed. Based on the clustering method Barcode Index Number (BIN) we found a taxonomic bias of approximately 30% in our initial morphological assessment. However, a morphological reassessment revealed that the correspondence between morphospecies and molecular operational taxonomic units (MOTUs) can be up to 94% if differences in genitalia morphology are evaluated in individuals of different MOTUs originated from the same morphospecies (putative cases of cryptic species), and by recording if individuals of different genders in different morphospecies merge together in the same MOTU (putative cases of sexual dimorphism). The results of two other clustering methods (i.e. Automatic Barcode Gap Discovery and 2% threshold) were very similar to those of the BIN approach. Using empirical data we have shown that DNA barcoding performed substantially better than the morphospecies approach, based on superficial morphology, to delimit species of a highly diverse moth taxon, and thus should be used in species inventories.

## Introduction

The immense work of cataloguing diversity of all arthropods on Earth remains one of the most challenging scientific ambitions of our day. In one of the most comprehensive attempts to record terrestrial arthropods, [1] Basset et al. estimated that a single hectare of rainforest in Panama is inhabited by 18,439 species. Biodiversity inventories of arthropods in different parts of the world also show high diversity patterns, with Coleoptera and Lepidoptera being among the most diverse groups, particularly in tropical rainforests, *e*.*g*. [2, 3]. The ability to reliably assign unknown individuals to species is critical in assessing arthropod biodiversity [4,5] and often represents a considerable challenge in tropical regions, requiring scarcely available expertise and important resources. In addition to the effects on spatial ecology and conservation, which are rarely assessed, spurious species identifications can lead to error cascades in many biological studies affecting the decision-making processes to control diseases [6], fight crop pests [7], and also to produce drugs and other bioactive compounds [8,9].

In tropical and subtropical environments, where arthropod biodiversity is usually high and taxonomy has been neglected, morphological species (morphospecies) are inevitably used as surrogates for species, *e*.*g*. [1, 10]. Under the morphospecies assumption, the sorter must recognize units without the use of taxonomic sources, relying exclusively on superficial morphological characteristics [11]. Although this appears to be a tempting approach different operators rarely reproduce the same results and the correspondence between morphospecies and taxonomic species can vary extensively, with error rates being higher than 100%, depending on which groups are considered [5]. As an alternative, DNA barcoding—a 658-bp fragment of the mitochondrial gene cytochrome c oxidase I (COI)–which has been widely used for more than a decade, shows a high correspondence with taxonomical identification, particularly for distinguishing arthropod species, *e*.*g*. [12–17]. This method has two advantages compared to the morphospecies approach: (1) two or more people can unambiguously assign individuals to species without requiring any taxonomic expertise; and (2) a species identification can be obtained if a barcode sequence is compared to sequences of conspecifics deposited in a database for which the taxonomic name is already known (*i*.*e*. DNA barcode library, [18]).

DNA barcoding has been applied to Lepidoptera diversity since the beginning of the barcoding initiative, with several campaigns in different parts of the world [19]. Although some studies have reported a low performance [20–23], mostly attributable to interspecific hybridization or endosymbiont infections, DNA barcoding is considered to be a highly effective tool for uncovering hidden diversity in Lepidoptera [14,24]. This success can be attributed in large part to the fact that most of these inventories are being undertaken to assemble DNA barcode libraries for well-known taxa, usually covering temperate regions [25–28]. The “Area de Conservación Guanacaste” inventory [29,30] was the first to reveal a substantial number of cryptic species as well as overlooked cases of sexual dimorphism using this method. Since then, similar studies have assessed local diversity of Lepidoptera in tropical and subtropical regions [21,31,32].

Although it is still impractical to assemble libraries of sequences covering large tropical and subtropical geographical areas, local inventories that couple traditional taxonomy with DNA barcoding can pinpoint taxonomic issues in species-rich, understudied lineages of Lepidoptera (e.g. noctuoids, geometroids), which in turn can foster the assembling of DNA barcode libraries for larger areas [29,30]. The development of DNA barcoding is not only uncovering a multitude of cryptic species that otherwise might not have been discovered, but it is also changing our knowledge of diversity patterns [33]. It has been shown that cryptic animal species are homogeneously distributed among taxa and biogeographical regions [34], and a recent study covering the western Mediterranean showed that cryptic species generate most butterfly beta-diversity [35]. Therefore, DNA barcoding and other molecular techniques are changing the way biodiversity is assessed, and whether applied to local inventories of species-rich taxa and regions various ecologically relevant discoveries might be made (see [36] for a review).

Moths in the subfamily Arctiinae (family Erebidae, superfamily Noctuoidea) are small to median-sized insects, often brightly colored. These aposematic colors warn predators of the moth’s un-palatability, acquired in some species through fixation within the larvae of pyrrolizidine alkaloids from the hostplant [37,38]. The subfamily is distributed worldwide with approximately 11,000 described species grouped into four tribes (i.e. Arctiini, Lithosiini, Syntomini and Amerilini) [39,40]. In the years 2010–2012, a biodiversity survey of arctiines with standardized field collections was undertaken in the highly threatened Atlantic Forest. The purpose of our project was to study elevational diversity patterns in a large remnant of this forest in southern Brazil, and compare those results with available data from other regions [41]. Additionally, during the course of our study almost all species found were barcoded and the resulting sequences were entered into the database of the Barcode of Life Data Systems—BOLD Systems—a worldwide database of DNA barcodes covering animals and other species [19].

The current project represents one of the largest inventories of moths using DNA barcoding in the Neotropical region. Neotropical noctuoid species assemblages are highly diverse [42,43], and thus the completion of a census of species, even in small areas, is highly challenging. Here, we sampled extensively over a two year period an area in the Atlantic Forest to produce a fast, though incomplete, census of arctiine moths whose species diversity was assessed using two approaches: (1) a superficial separation into morphospecies upon arrival in the laboratory and (2) separation into species units through the analysis of DNA barcodes. We used a recently proposed clustering method to delimit Molecular Operational Taxonomic Units (MOTUs) based on barcode sequences and we compared the correspondence of these MOTUs to morphospecies delimited based on wing and body pattern phenotypes. In the discordant cases, morphology was carefully re-evaluated by examining male genitalia and the results were compared to MOTUs delimited with three different automated methods of species delimitations. We highlight the performance of DNA taxonomy in assessing biodiversity of moths compared to the superficial morphological approach, although we suggest that additional data, such as life-history traits and nuclear genes, should also be incorporated for a more accurate delineation of species boundaries, especially in putative cases of cryptic diversity.

## Materials and Methods

### Study area and sampling method

The study area is located in southern Brazil, Paraná State, along a slope of Atlantic Forest ranging from 7 to 927 m a.s.l. The area is part of “Área de Proteção Ambiental da Serra do Mar” which is under the management and protection of Paraná State Environmental Institute (IAP, www.iap.pr.gov.br/). A total of 14 sites were sampled along the slope, in a transect line spanning approximately 15 km. Most of the sampling sites were within undisturbed primary forest, but some sites were located in slightly disturbed secondary forest. Sampling was done one night per month in nine sampling sites from February, 2010, to January, 2012, and sporadically in the remaining sites. The moths were collected using UV automatic light traps (Pennsylvania model, [44]) equipped with tubular lamps Sylvania model F15 T12 LN, 15 Watts. A plastic bucket filled with two liters of 95% ethanol was used as a killing jar at the base of the trap. All arthropods attracted to light traps were collected, but only arctiines were sorted. The remaining vouchers were stored in hermetic containers filled with 95% ethanol. See [45,41] for further details about sampling sites, trap design and sampling effort, as well as aerial images of the sampling area. The field collection permits were granted to JAT by the “Instituto Brasileiro do Meio Ambiente e dos Recursos Naturais Renováveis” and to MMZ by the “Instituto Ambiental do Paraná”. Those permits were issued by the national and state environmental authorities, respectively, and thus meet all the requirements of Brazilian environmental regulatory rules. No endangered or protected moth species were collected during the field work.

### Species delimitation methods

**Fig 1:**
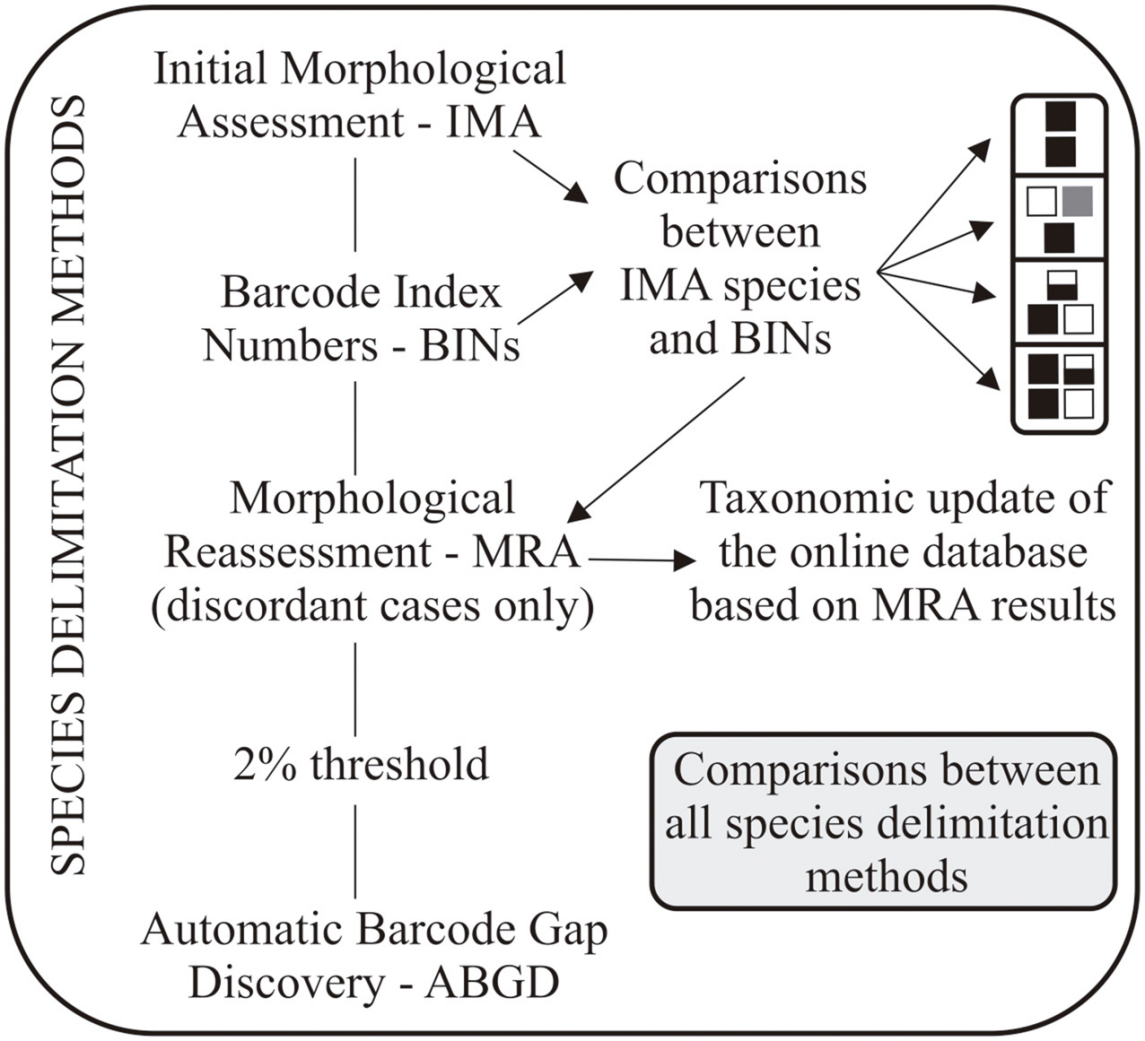
Schematic representation of the integrative species delimitation method used to uncover Tiger Moth diversity along an elevational gradient in southern Brazilian Atlantic Forest. Small black, white and grey squares in the upper right corner represent the possible rearrangements of the IMA species suggested by the analysis of the Barcode Index Numbers.

For the initial delimitation of species boundaries we chose the two fastest methods available: (1) we sorted the species in a superficial morphology-based method (Initial Morphological Assessment, from now on IMA) and (2) we delimited molecular operational taxonomic units (MOTUs) defined according to the Barcode Index Number system (from now on BIN) as implemented in BOLD. In a second step, we (1) reassessed morphology in cases where the IMA and BINs did not match (Morphological Reassessment, from now on MRA), and (2) we also reassessed MOTUs using two alternative automated species delimitation methods (i.e. 2% threshold and Automatic Barcode Gap Discovery (ABGD)). These new results were then compared to the IMA and the MOTUs defined by BINs (Fig 1).

The IMA was done along a two-year sampling period and all specimens were deposited at “Museu de Zoologia Adão José Cardoso” of “Universidade Estadual de Campinas”, Brazil. Almost every month “new” IMA species were recorded and, when available, at least three specimens were spread, pinned and photographed in dorsal and ventral views [45]. The IMA species delimitations were made independently by MMZ, based on examination of vouchers, and by JAT and ML based on voucher’ dorsal and ventral digital images. Only external morphology was considered (i.e. color and drawing patterns of wings, head, thorax, abdomen and legs); genitalia morphology or other internal structures were not assessed during the IMA. When the species delimitations did not match, one of them was chosen by MMZ based on comparisons between vouchers. Heavily damaged specimens that could not be reliably assigned to species were discarded. The species were identified by JAT based on references cited in [46] and by ML based on his private collection and references cited in [47–49]. When a species identification was not possible the genus name was followed by the identifier initials and a number (e.g. *Trichromia* sp. JAT04, *Scaptius* sp. ML01), and when a species was delimited but the genus name was unknown it was identified by the subfamily name, followed by the delimiter initials and a number (e.g. Arctiinae sp. JAT05).

The MOTUs were defined based on standard DNA barcoding for animals [12,18]. When an IMA species received a name tissue samples were removed from vouchers and shipped to Canadian Centre for DNA Barcoding (CCDB), along with the specimen’s identification and collection data. To represent intraspecific variation at least three vouchers of each species were used, except in the cases of species where only one or two specimens were collected (i.e. singletons and doubletons). The samples and the data were shipped periodically from March, 2010, to December, 2011. All sequences used in this work were generated by the CCDB following standard protocols for extraction, PCR amplification, product checking and sequencing described in the CCDB web site [50]. We built a database (identified by its project code LEMMZ) hosted online by the Barcode of Life Data systems (BOLD) [19], where each specimen is represented by a record combining taxonomic information, collection data, sequence data (including trace files), as well as digital images in dorsal and ventral views.

The Barcode Index Number is an interim taxonomic system used by the BOLD Systems, based on refined single linkage algorithm (RESL), to cluster DNA barcode sequence data into MOTUs [51]. This system has been proved to be highly efficient to cluster barcode sequences of large datasets of Lepidoptera species and outperforms all available methods based on different algorithms [51]. Once a barcode sequence enters the BOLD database it is automatically assigned to a BIN, which may contain similar sequences available in the database [52]. We therefore compiled the taxonomic names within each BIN obtained exclusively from the specimens we found in our study area in the Atlantic Forest whose barcode sequences are deposited in the BOLD database. The names of the sequences of other studies that eventually may have fall into BINs shared with our sequences were disregarded.

The MRA was restricted to the conflicting results between IMA and BINs. Those cases of agreement between IMA and BINs species delimitations were treated as well-delimited species and were posteriorly computed as being MRA species. Three basic outcomes may arise when the IMA species do not match BINs: an IMA species splits into two BINs (splits); two IMA species merge into the same BIN (merges); and two individuals from the same IMA species split into different BINs and at least one of them merges with another individual from a different IMA species (mixtures)–see Fig 1. However, it is worth noting that these three outcomes, especially the mixture cases, can vary a lot with up to three IMA species merging and two or even three cases of splits, as shown elsewhere [29,32,51]. Initially, the IMA species that did not match BINs were sorted into these three basic outcomes, and their variations, and all individuals had their sex determined. We recognize several causes for incorrectly delimited IMA species. Two of these, with opposite effects on appraisal of species diversity, are overlooked cryptic species [29,32] (very similar or even identical wing-pattern shared by distinct species) and intraspecific polymorphism where variants of a same species are erroneously considered heterospecific. Sexual dimorphism (male and females being so different that they are incorrectly assigned to distinct species) is a special case of the later, and was shown to be possibly spectacular in arctiine moths [see *e*.*g*. 53] and then of primary relevance here. Other causes for IMA/BIN mismatches include operational biases like contamination events or human errors in databasing/sorting of samples, or biological causes like very recent speciation events and incomplete lineage sorting, introgression and hybridization, manipulation of reproductive systems by endosymbiotic bacteria. None of these were directly tested here (*f*.*i*. through reprocessing of specimens, or adding nuclear markers, testing for *Wolbachia* infection and strains, etc.), and they will remain as possible explanations when morphological reassessment does not affect the delimitation of IMA species.

Here, the genitalia morphology was compared between individuals within the same BIN if it was originated from different IMA species, and between different BINs if they were originated from the same IMA species. In most of cases the comparisons were made with males but females were used in a few cases. Only visual morphological characteristics were used and no morphometric analysis was done.

After the morphological reassessment based on BINs was concluded the species delimitations were reorganized accordingly and the taxonomic identifications were improved based on comparisons made with vouchers deposited in the Vitor O. Becker collection [54]. The specimens were compared based exclusively on habitus since vouchers were not allowed to be dissected. To distinguish the putative cryptic species delimited in this study the letters MMZ and a number were added after the species name (e.g. *Cosmosoma auge* sp. MMZ01, *Eucereon rosa* sp. MMZ02). Finally, we used all information provided by our integrative species delimitation method and the new taxonomical data to update the online database.

The same set of barcode sequences assigned to BINs was used to delimit species within a 2% threshold, and with the ABGD method. Similarly to the BIN system these two methods are commonly used and have been proved to be efficient for clustering DNA barcode sequences of Lepidoptera into MOTUs [13,55]. A previous study showed that the results of different methods can be different depending on the taxonomic group [51], and thus the comparisons made here may help to evaluate different clustering methods. The degree of sequence divergence between two samples above a given threshold indicates specific distinctness [22]. Thus, the sequences were assigned into MOTUs if the genetic distance between sequences was lower than 2% (2% threshold). The ABGD method calculates distances among all pairs of sequences and clusters them by creating a division at points where the change in slope of the distribution is highest. Partitions are recursively evaluated for division points, and splitting is sustained until all partitions possess a unimodal distribution [55,51]. The pairwise distances between sequences were calculated with Mega 6 [56], and the Automatic Barcode Gap Discovery was implemented online with the tool abgd web [55]. Finally, we compared the number of shared species in each possible comparison between species delimitations methods to evaluate their relative performance.

## Results

The IMA species delimitations in the present study were highly concordant among authors, except for a few cases in which some individuals that were clearly conspecifics were erroneously assigned to different species. These discrepancies were resolved after the collection was completed and a final revision on species delimitations was done. A species list with all 291 IMA species is provided in the S1 Appendix. A total of 158 IMA species were identified to species level, and 97 and 36 IMA species were identified to genus and subfamily level only, respectively. DNA extraction and sequencing were successful for most samples, and only 17 failures were recorded, including samples of four species cited in Zenker et al. [41] that were not included in the analysis. The mean sequence length of the 1,100 barcode sequences (Arctiinae only) in our project in the BOLD Systems was 653 bp ± 2.3 SD, and the mean number of sequences per IMA species was 4.43 ± 2.7 SD, excluding singletons. No contamination or stop codons were detected. The GenBank accession numbers and BOLD sample IDs of all sequences used in this study are available in the S2 Appendix, and are publicly accessible from GenBank and BOLD database, respectively.

### The Barcode Index Number approach

**Fig 2:**
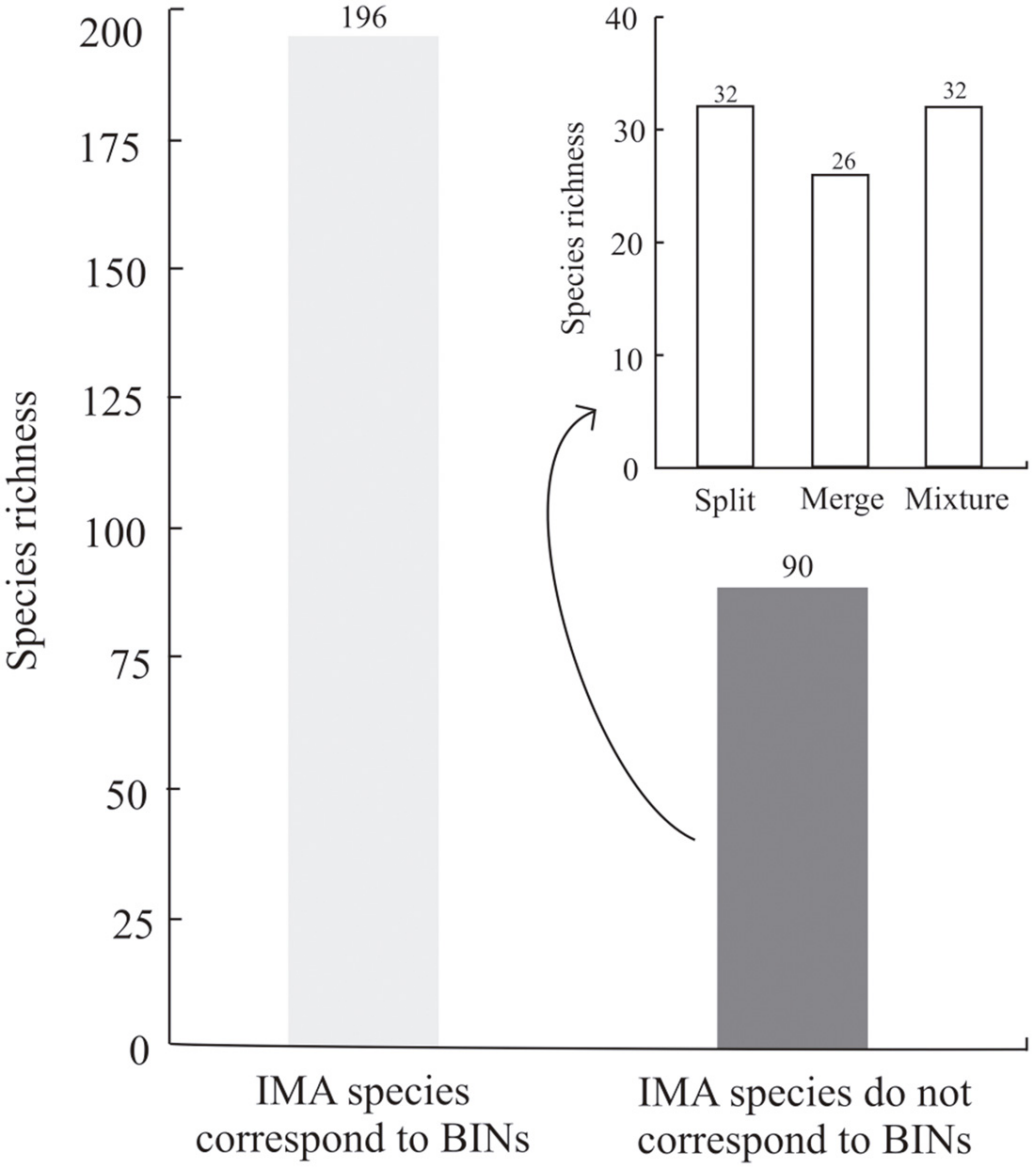
Correspondence between species delimited according to an Initial Morphological Assessment (IMA) and molecular operational taxonomic units delimited with the Barcode Index Number approach (BINs). The inset figure shows the distribution of the rearrangements in the IMA species, as suggested by the BINs analysis.

**Fig 3:**
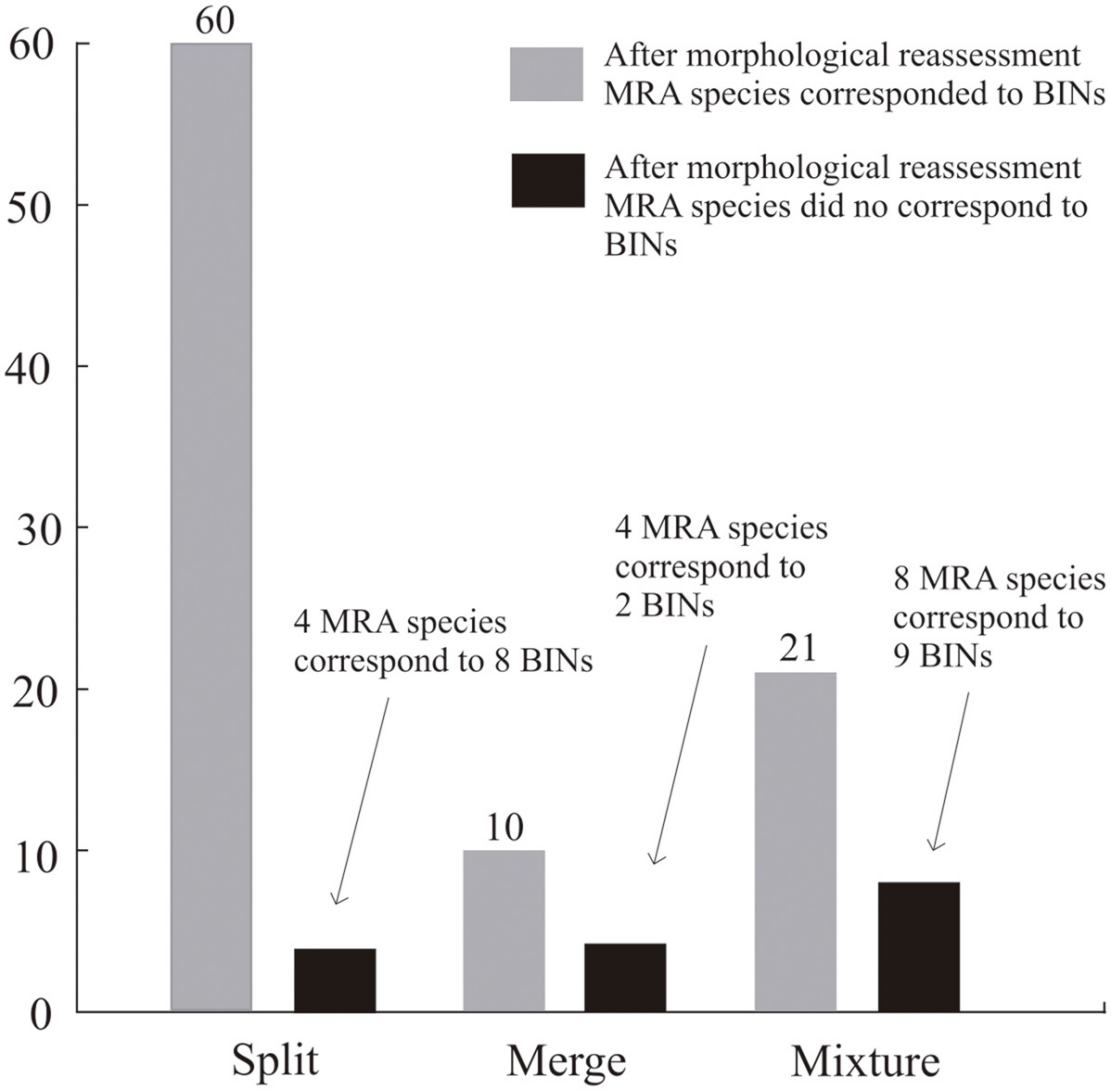
Results of the Morphological Reassessment (MRA) to assess incongruences between species delimited according to the IMA and BIN systems.

A total of 1,075 barcode sequences were assigned to 306 BINs (Table 1), excluding 25 sequences of five IMA species. Those excluded species could not be analyzed morphologically, because individuals within each BIN were of different sexes (see S1 Appendix), thus precluding direct comparison of the specimens. This represents an increase in the species richness of the initial morphological assessment of nearly 6.5%. A list of the BINs and the IMA species therein contained is provided in the S1 Appendix. From a total of 286 IMA species analyzed, 196 species corresponded to BINs (Fig 2 larger graph, Table 1), and therefore their morphology was not reassessed (see material and methods section), but in 90 species morphology had to be reassessed because the IMA species delimitations did not correspond to BINs (Fig 2 larger graph). The inset in Fig 2 shows the distribution of the rearrangements in the IMA species that did not correspond to BINs. A total of 32 of the 90 IMA species without correspondence to BINs split into 68 BINs, of which 60 were confirmed to correspond to MRA species after the morphological reassessment; the remaining eight BINs corresponded to four MRA species (Fig 3). A total of 26 IMA species merged into 12 BINs, of which 10 were confirmed to correspond to MRA species; the remaining two BINs corresponded to four MRA species (Fig 3). And finally, 32 IMA species mixed into 30 BINs, of which 21 were confirmed to correspond to MRA species; the remaining nine BINs corresponded to eight MRA species (Fig 3). A total of 303 MRA species were recorded in this study, including 196 IMA species that corresponded to BINs and additional 107 MRA species delimited after the morphological reassessment. The number of species corresponding to BINs increased from 196 according to the initial morphological assessment to 287 after the morphological reassessment (Table 1).

**Table 1:** Pairwise comparisons of the shared number of species of an assemblage of Tiger Moths recorded along an elevational gradient in southern Brazilian Atlantic Forest delimited with five different species delimitation methods. IMA: Initial Morphological Assessment; MRA: Morphological Reassessment; BINs: Barcode Index Numbers; 2% threshold approach; ABGD: Automatic Barcode Gap Discovery. MOTUs, BINs and S are generically referred as to species.

	IMA	MRA	BINs	2% threshold	ABGD
	S = 286	S = 303	BINs = 306	MOTUs = 302	MOTUs = 304
IMA	–				
MRA	207	–			
BINs	196	287	–		
2% threshold	199	285	298	–	
ABGD	197	283	296	299	–

After the morphological reassessment the species identifications were revised (see Material and Methods section) and a total of 253 species were identified to species level, including 56 cases of putative cryptic species originated from the rearrangements among conspecifics or congeners that were identified by the species name followed by the initials MMZ (*e*.*g*. *Cosmosoma auge* sp. MMZ01, MMZ02 etc.); 37 and 18 species were identified to genus and subfamily level only, respectively. All taxonomic data obtained was uploaded into the online database. A list of the MRA species and their respective BINs and IMA species is provided in the S1 Appendix.

### Comparisons between different approaches

**Fig 4:**
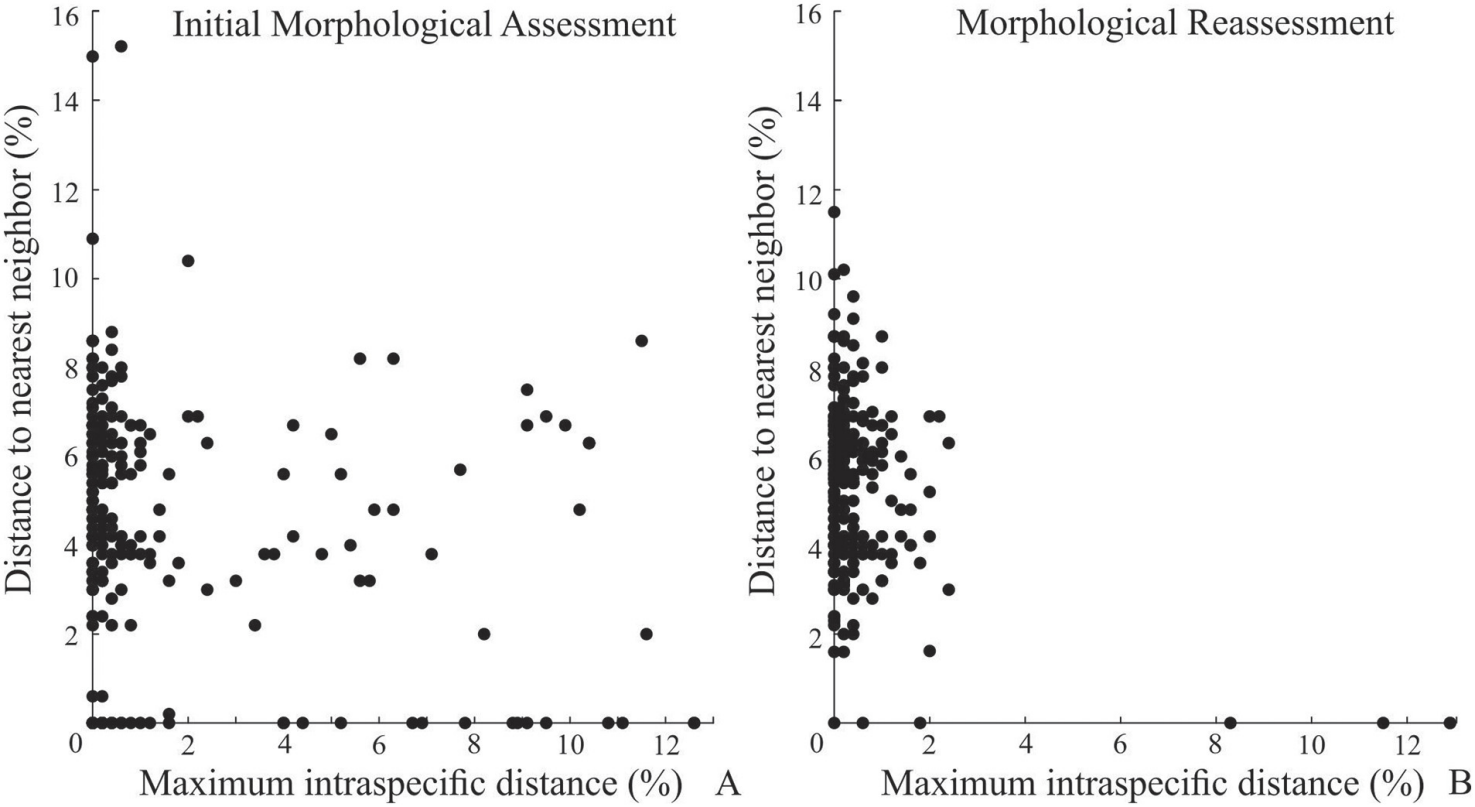
Maximum intraspecific distance and distance to the nearest neighbor of the 157 species with two or more sequences delimited under the Initial Morphological Assessment (A) and Morphological Reassessment (B) approaches.

**Fig 5:**
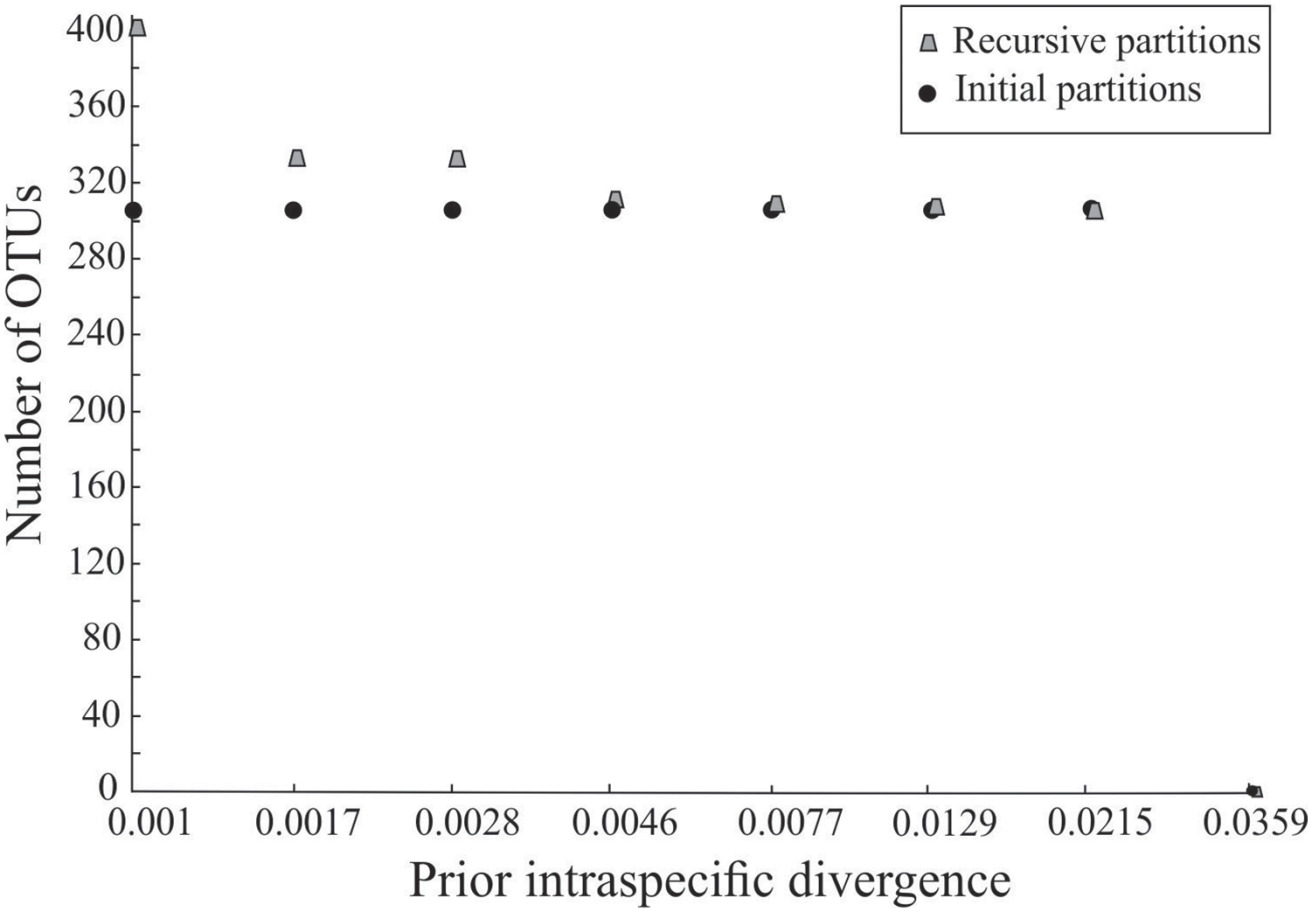
Recursive and Initial partitions delimited with the Automatic Barcode Gap Discovery approach (ABGD) used to uncover Tiger Moths diversity along an elevational gradient in southern Brazilian Atlantic Forest.

A total of 302 MOTUs were delimited under a 2% approach with the same set of sequences used to delimit BINs (Table 1), representing an increase in the species richness of the initial morphological assessment of nearly 5.6%. A total of 199 MOTUs delimited with a 2% threshold analysis corresponded to IMA species (Table 1), including 42 singletons and 157 IMA species with two or more sequences (Fig 4A). After the morphological reassessment a total of 285 MOTUs delimited with a 2% threshold analysis corresponded to MRA species (Table 1), including 61 singletons and 224 MRA species with two or more sequences (Fig 4B). The ABGD approach revealed that a total of 304 MOTUs were found with prior interspecific divergence of 0.0215 (Fig 5), representing an increase in the species richness of the initial morphological assessment of nearly 6.3%. A total of 197 species delimited under the ABGD approach corresponded to IMA species (Table 1).

The number of MOTUs corresponding to IMA species was very similar between species delimitation methods (Table 1). However, the ABGD approach showed that two IMA species (*Baritius acuminata* and *Cosmosoma theutras*) split into two MOTUs while the 2% threshold and BIN approaches showed only one. After the morphological reassessment, however, these two MOTUs were not confirmed to be MRA species (see S1 Appendix). Differently from the ABGD and 2% threshold approaches the BIN system showed that the IMA species *Delphyre* sp. ML01 split into two MOTUs, which were confirmed to be MRA species and then renamed to *Prosopidia oviplaga* sp. MMZ01 and MMZ02 after the taxonomic identifications were improved (see S1 Appendix). In addition, the BIN system differed from the other molecular species delimitation methods in three other IMA species (*Phaegoptera fusca*, *Leucanopsis leucanina* and *Melese chozeba*). The first species split into three BINs and the other into two but none of them was confirmed to be an MRA species (see S1 Appendix).

The number of MOTUs corresponding to MRA species was also similar between species delimitation methods. From a total of 303 MRA species, 287 could be separated into BINs, 285 into MOTUs under a 2% threshold approach (see Fig 4B for an illustration without singletons) and 283 into MOTUs according to the ABGD approach (Table 1). Therefore, the performance of the three species delimitation methods to separate morphological species varied within a range of slightly more than 1%. Similarly, the MOTUs delimited with the three molecular species delimitation methods were very similar, with discordances also only found in slightly more than 1% (Table 1) of all MOTUs.

## Discussion

This project (Lepidoptera of Serra do Mar—LEMMZ) is among the ten largest projects in BOLD with respect to the number of sequences of arctiine moths. It accounts for approximately 75% of DNA barcode sequences of this taxon recorded from Brazil (data accessed in June 15^th^ 2015). Two factors were important in obtaining a large volume of high quality sequences. First, fresh specimens were used preventing DNA degradation and second, the optimized protocols developed by the CCDB [50,57] resulted in a large amount of DNA extracts and high quality PCR products. Although the current protocols allow DNA extraction of 30 or more years old museum specimens, the sequences frequently do not exceed 500 bp and might not be able to differentiate species in large assemblages [13,24]. However, in more than 96% of the sequenced specimens in our project the sequences were 658 bp long and virtually all species could be distinguished. In addition, the surplus DNA extracted can be used to obtain nuclear sequences that could be used to back up barcode results and to access phylogenetic information, as shown elsewhere [14,58].

Our integrative species delimitation method revealed that approximately 30% of the species studied were initially incorrectly assigned to morphospecies. This was grounded on different morphological and molecular species delimitation approaches. Sexual dimorphism is one of the most important, possibly confusing, sources of intraspecific variation in the Lepidoptera [59]. One of the most obvious explanations for most of the cases of merges, and a few cases of mixtures, found in our study is the occurrence of sexual dimorphism. Without previous knowledge of gender’s appearance it would had been impossible to find cases of sexual dimorphism in our fast census of arctiines without the aid of barcoding, especially if we consider that other sources of intraspecific variation such as mimicry and polymorphism could have been mistaken with sexual dimorphism [60]. A long term inventory of arctiines in our study area could link genders of sexually dimorphic species but such a research could last for years or even decades, and depending on the sampling effort would be too expensive to be undertaken. Thus, DNA barcoding is revealed to be a highly valuable tool to link genders of sexually dimorphic species in our Lepidoptera biodiversity research, as previously shown elsewhere [29,30], and in our opinion should be used routinely in every species inventories, especially in countries in short of taxonomists.

The lock-and-key hypothesis, which purports to explain species-specific genitalic morphology in terms of mechanical reproductive isolation, dates back to the 19^th^ century [61]. Several authors have argued that morphology of male genitalia is more variable than female and because of that it would be possible to conspecific females to mate with males of different species (see a review in Shapiro and Porter [62]). Mating tests showed that interspecific, intergeneric and even interfamilial hybridization in Lepidoptera is possible and that the precise configuration of the male armature is not a precondition for mating success [63]. However, many species descriptions in Lepidoptera are based primarily on morphological characters of male (occasionally female) genitalia, the morphology of which tends to reveal clear-cut species-specific diagnostic characters with much higher frequency than in other body structures [62]. Although a visual evaluation of genitalia morphology may also be seen as preliminary compared to modern morphometric techniques [64], the morphological differences we found were conspicuous, and many of the cases of DNA barcode splits, as well as a few cases of mixtures (see S1 Appendix) represent unequivocal cases of overlooked cryptic species which will be formally described in the future. Eventually we observed only few cases where differences were subtle and somewhat equivocal, suggesting the need for a thorough morphometric analysis of morphological variations and the possible investigation of other sets of characters such as behavioral and/or ecological features [65].

Although the presence of cryptic species and sexual dimorphism are reasonable explanations to explain most cases of splits, merges and mixtures, other causes might be involved, such as incomplete lineage sorting, genetic introgression from closely related species [66,67] or occasional hybridization with other species [68]. Alternatively, reproductive isolation within species can be impacted by endosymbiont infections by *Wolbachia* [69,70] causing cytoplasmic incompatibilities and the persistence within populations of divergent mitochondrial lineages [71, 72]. Although we did not test whether *Wolbachia* infection, mtDNA introgression and/or incomplete lineage sorting affected our results, we considered that in all revealed cases of overlooked sexual dimorphism, this special case of intraspecific polymorphism represented the obvious most likely explanation, often supported by the occurrence at the same site of specimens of distinct sexes sharing DNA barcodes.

Another factor that might have affected the initial assignment of individuals into morphospecies was the type of light trap used, which can cause damages to the specimens captures, thus affecting the operator’s ability to properly sort and assign individuals to morphospecies. Light trap monitoring programs have been undertaken in several places, most notably in Great Britain where the Rothamsted Insect Survey has been active uninterruptedly for more than 50 years [73]. Although the Rothamsted and Pearson designs are considered highly efficient, a plethora of light traps have been used to study moth diversity [74] and no standard design is universally used. Here, we chose the Pennsylvania light trap because our initial goal was to compare diversity patterns found in our study area with available data obtained using the same type of trap in the southernmost limit of the Atlantic Forest [41]. In our experience this type of light trap works well for the noctuoids [75,76], with specimens collected in good condition, contrary to the frequent erroneous assumption that they would be too damaged for proper separation of species. We were not able to find any published work comparing the effectiveness of morphospecies delimitations of arctiines using different kinds of light traps, but the type of trap we used might in fact have had an impact on our initial morphological assessment. Interestingly, DNA barcoding revealed cases in which obvious morphological differences between specimens in the same IMA species passed unnoticed in the initial morphological assessment, as shown elsewhere [29], but also revealed cases of slight differences between specimens in the same IMA species that would have required the analysis of series of specimens in perfect conditions, and typical cases of cryptic species which are impossible to distinguish, even through comparison of specimens in perfect conditions. It is also worth noting that our results, revealing a more than 94% match between MRA species and BINs, were not affected by contamination issues caused by detached scales of specimens caught in ethanol. However, 16 MRA species could not be separated using BINs, and although it may be caused by a number of factors (see [77]), as previously discussed, we could not discard the possibility of contamination without further testing. In summary, the Pennsylvania light trap allowed a reliable preliminary assessment of morphospecies diversity in our study, which was improved with barcoding technology and confirmed with dissections of the specimens.

Several clustering methodologies using different kinds of algorithms have been recently developed, such as ABGD [55], BINs [51], and GMYC [78], and we have rigorously compared refined morphological species delimitations to the results of three of those approaches. One clear outcome of this work is that the MOTUs delimitations did not differ considerably between the three established analytical approaches used (Table 1). The Barcode Index Number system performed slightly better than the other methods, revealing only one additional putative cryptic species that was backed up by genitalia morphology (see Results section). Therefore, our empirical evidence supports the use of the BIN system for automated species delimitation of Arctiinae moths in local inventories.

In the last decade, Internet-based projects has allowed access to Lepidoptera biodiversity information and one of the most famous example is the “Area de Conservación Guanacaste—ACG” project, in Costa Rica [29,30]. In this biodiversity inventory, almost 200,000 caterpillars of 2,810 morphospecies originated from the several habitats within ACG were reared and all life stages and host plants documented in an online database [79]. This project also was a pioneer in evaluating DNA barcoding as a tool to reveal hidden diversity in the Lepidoptera and it is the largest in the BOLD Systems in number of sequences (data accessed in June 15^th^ 2015). Similarly to our project, the ACG project revealed several cases of putative cryptic species and sexual dimorphism when the cases of splits, merges and mixtures were closely analyzed. However, one of the clearest outcomes of the ACG study is that the number of individuals for each species must be large (at least 20 specimens). This is because even in a “good” species with four or five individuals showing no or little intraspecific genetic divergence at the DNA barcode locus, cryptic species can be detected if more sequences are added, especially if those specimens originated from different habitats and/or distant regions [29,30]. This observation should bring caution when considering the results of our survey, because most species are represented by two to nine sequenced specimens and several by singletons only. Therefore, the species richness revealed here using DNA barcoding may be an underestimate, and the number of species may increase with additional sampling and when more sequences are added. Another important aspect of the ACG inventory was the possibility to correlate barcode clustering with habitat, behavior and immature stages morphology. Indeed, the ACG inventory has been ongoing for 50 years and more than 150 members of taxasphere (including 50 professional taxonomists) contributed to its development [29,30] while ours is a much smaller project that employed two taxonomists. Nevertheless, the information here reported will certainly be useful to the development of a database similar to that of the ACG project that may be used not only by biodiversity scientists working in our study area but also in other regions of the Atlantic Forest.

Although our project is far from representing a comprehensive library of DNA barcodes, it is expected to be useful to ecological studies and the understanding of insect-plant relationship in the Atlantic Forest. Based on this library many species of arctiines of the Atlantic Forest can be reliably identified and the host-plant could be linked to the adult if a single caterpillar found feeding in a tree is barcoded, as ecemplified elsewhere [29,32]. Also, the diet analysis of the insectivorous predators can be assessed by barcoding the stomach contents of bats, birds, small mammals, frogs, lizards and other predators [36]. Indeed, the Bold Systems database has been used successfully to reveal the diet of bats in Canada [80] and Finland [81]. Therefore, we believe that our contribution to the library of DNA barcodes in arctiine moths will be of great help for the development of many other biological studies in the Atlantic forest in the future.

The success of biodiversity inventories of moths and other hyperdiverse arthropod taxa rely strongly on comparisons with specimens deposited in natural history collections, and thus taxonomy must be very accurate. Despite the efforts made by the available taxonomists a much higher number of professional taxonomists would be required to describe all biodiversity on earth before it disappears due to loss of habitats [12], and thus alternatives must be considered. By comparing DNA barcoding results with genitalia morphology we have shown that barcoding can be used as a taxonomical tool in inventories of species-rich taxa and can help to foster descriptions of new species.

## Supporting Information

S1 Appendix List of species recorded along an elevational gradient in southern Brazilian Atlantic Forest delimited according to five different species delimitation methods. For a description of species delimitation methods see Material and Methods section. Cells with colors of different shades are used to separate BINs. Cells in different shades of green: matching results between all five species delimitation methods; Blue cells: conflicting results between the ABGD method and the remaining approaches. Cells in different shades of yellow: matching results between the molecular approaches (BINs, 2% threshold and ABGD) and the MRA species delimitation method but neither the molecular nor the MRA approaches corresponded to the IMA species delimitation method. Cells in different shades of purple: matching results between BINs and MRA species delimitation method, but conflicting results between these two methods and the remaining approaches (IMA, 2% threshold and ABGD). Cells in different shades of red: conflicting result between the molecular species delimitation methods and the remaining approaches. IMA and MRA species delimitation methods may or may not correspond to each other (see rearrangements into MRA species). Cells in different shades of brown: conflicting results between BINs and the remaining molecular species delimitation methods. IMA and MRA species delimitation methods may or may not correspond to each other (see rearrangements into MRA species). The species names may differ because taxonomy was improved after the morphological reassessment (see Material and Methods section).
(XLSX)

S2 Appendix Accession number of the sequences used in this study. (XLS)
